# Advances in Traditional Chinese Medicine interventions for influenza A based on the gut-lung axis: modern evidence for the exterior-interior relationship between the lung and large intestine

**DOI:** 10.3389/fmed.2026.1766424

**Published:** 2026-02-25

**Authors:** Qiong Chen, Jie Gong, Hongming Fan, Guobin Tao, Chaoliang Lin, Xiangjin Cheng, Honglin Zhu

**Affiliations:** Department of Emergency, Yancheng TCM Hospital Affiliated to Nanjing University of Chinese Medicine, Yancheng, Jiangsu, China

**Keywords:** active ingredients, Chinese herbal formulas, gut microbiota, gut-lung axis, influenza A virus, polysaccharides, Traditional Chinese Medicine

## Abstract

Influenza A virus (IAV) is a common respiratory pathogen that causes seasonal epidemics and severe infections, imposing substantial healthcare and economic burdens. IAV infection can disrupt the gut microbiota, and the resulting dysbiosis influences host status during both the early and later stages of infection. In recent years, Traditional Chinese Medicine (TCM) has shown considerable potential in the treatment of IAV. Guided by the TCM concept of the exterior-interior relationship between the lung and large intestine and integrated with contemporary research on the gut-lung axis, this review summarizes advances in the mechanistic and preclinical evidence of Chinese herbal formulas, individual compounds, and polysaccharides in influenza A and outlines mechanism-based research directions and translational insights to inform future exploration.

## Graphical Abstract

**Figure:**
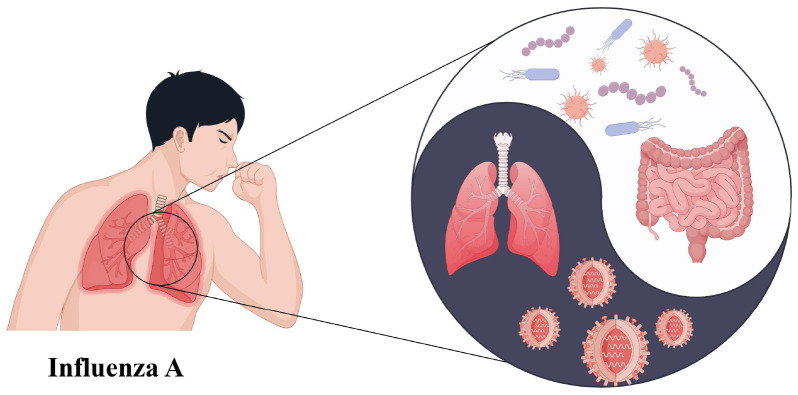
Traditional Chinese Medicine (TCM) modulation of the gut-lung axis to intervene in influenza A.

##  1 Introduction

Influenza is an acute respiratory infectious disease caused by influenza virus, characterized by fever, sore throat, rhinorrhea, cough, headache, myalgia and arthralgia, and profound malaise (1). Some patients present with gastrointestinal symptoms such as diarrhea and vomiting. High - risk groups are prone to progression to severe influenza, manifested as rapidly progressive interstitial pneumonia; in severe cases, acute respiratory distress syndrome and multiple organ dysfunction syndrome may occur

(2). Clinically, H1N1 is the most common subtype of influenza A virus (IAV). Its hallmark is annual seasonal epidemics, and the severity of pandemics largely depends on the virulence of the strain and pre-existing immunity (3). Epidemiological studies indicate that approximately one billion people worldwide are infected with IAV each year, resulting in hundreds of thousands of deaths and imposing a substantial medical and social burden (4). Vaccination is the most effective method to prevent influenza A (5, 6). Clinically, the main treatment strategies include early diagnosis, antiviral therapy (7), immune enhancement (8), and management of complications. However, due to antigenic drift and antigenic shift in IAV, mismatches can occur between circulating strains and vaccine strains, compounded by interindividual heterogeneity in immune responses. Moreover, antiviral drugs have a narrow window for administration, and long-term use is prone to resistance and adverse effects, underscoring the urgent need for new therapeutic approaches (9).

The gut-lung axis refers to bidirectional cross-talk between the gut and respiratory tract mediated through interactions involving the microbiota and host immune functions (10). In clinical applications, research on modulating the gut-lung axis has become a focal area and may offer new avenues and strategies for influenza prevention and control (11). Recent studies suggest that Traditional Chinese Medicine (TCM), characterized by multi - component, multi - target, and multi - pathway actions, can effectively modulate the gut microbiota, thereby enhancing the immunogenicity and protective efficacy of influenza vaccines and attenuating host inflammatory responses, ultimately ameliorating IAV - related clinical symptoms.

This is a narrative, mechanism-focused synthesis. We searched PubMed/MEDLINE, Web of Science, Embase, and Chinese databases (CNKI, Wanfang, VIP) for relevant literature from 1 January 2014 to 1 December 2025. Representative search terms included: influenza A, IAV, H1N1; gut-lung axis, intestinal flora, microbiota, microbiome; traditional Chinese medicine, TCM, Chinese herbal formula, decoction; polysaccharide, ginsenoside, berberine, andrographolide, baicalein, etc. Articles were selected for relevance to mechanistic and preclinical evidence on TCM interventions for IAV; clinical endpoints were not systematically assessed. This review aims to extend existing work in two areas. First, it proposes an integrated clinical and mechanistic framework that bridges the TCM exterior-interior theory with the modern gut-lung axis, using TCM pattern stratification to operationalize “treating the lung via the intestine” across wind–heat invasion, heat–toxicity attacking the lung, toxic heat obstructing the lung, damp–heat in the intestine, and damp–heat in the lung and spleen. Second, it synthesizes evidence across formulas, single compounds, and polysaccharides, and, informed by multi-omics and signaling pathways, systematically summarizes common microbe–metabolite-immune mechanisms. The focus is on mechanistic and preclinical evidence; clinical endpoints are not systematically assessed in this review.

## 2 Coupled regulation among microbiota, metabolites and immunity in IAV therapy

The gut and lung engage in bidirectional communication and mutual influence through immune, metabolic, and neural pathways; the health status of the gut microbiota affects pulmonary immune homeostasis and disease susceptibility, and vice versa. In the context of respiratory viral infections such as IAV, gut dysbiosis and disruption of internal homeostasis often occur in parallel and are associated with disease severity and duration (12). However, there is complex bidirectional causality between “infection causing dysbiosis” and “microbiota state influencing infection outcomes.” Viral replication, host inflammatory responses, dietary changes, and the use of antibiotics, antivirals, proton pump inhibitors, and other medications can simultaneously affect the microbiome and clinical outcomes, complicating attribution. Influenza - related studies show that the gut microbiota can influence the occurrence and progression of respiratory diseases (13) and modulate host innate and adaptive immunity against IAV (14). Heterogeneity across studies in microbiota compositional changes and immune phenotypes suggests that sampling time points, sequencing strategies, diet, and drug exposures may affect results, necessitating tighter control in more standardized designs. After influenza infection, the abundance of *Bacteroidetes* increases while Firmicutes decreases, overall diversity changes are limited, and patterns are influenced by viral type (15). In an H1N1 setting, antibiotic intervention reduces the abundance of several gut bacterial families, coinciding with upregulation of AP-1/NR4A inflammatory pathways, increased dendritic cell activation, and impairment of H1N1 - specific neutralizing IgG1 and IgA responses (16). Metagenomics and targeted metabolomics indicate that indole-3-propionic acid in mouse blood is significantly reduced at day 7 post-IAV infection; supplementation lowers viral load and alleviates pulmonary and systemic inflammation, suggesting it is a gut - derived protective factor with anti-IAV activity (17). Additionally, IAV can concomitantly damage lung and intestinal tissues, disrupt gut microbiota and metabolites, and affect the Th17/Treg balance; supplementation with tryptophan and *Lactobacillus* helps ameliorate these phenotypes (18). Segmented filamentous bacteria can suppress IAV - induced proinflammatory gene expression in alveolar macrophages and enhance their viral-inactivation capacity (19). *Bifidobacterium pseudocatenulatum* NjM1 produces acetate and, via the GPR43-NLRP3-MAVS-type I IFN signaling axis, augments anti-IAV immunity, indicating that short-chain fatty acids and their receptor pathways may have therapeutic value (20).

These findings suggest that coupled regulation among microbiota, metabolites, and immunity is likely the key foundation for the gut-lung axis to exert its effects during IAV infection. Preclinical studies converge on several microbial and metabolic nodes. Beneficial taxa frequently increased by TCM or associated with protection include *Akkermansia*, *Lactobacillus*, *Faecalibacterium*, *Bifidobacterium*, and SCFA-producing *Lachnospiraceae*/*Ruminococcaceae*, whereas pathobionts such as Proteobacteria and Helicobacter are often reduced. Among metabolites, short-chain fatty acids and indole derivatives are repeatedly implicated: acetate can enhance antiviral responses via the GPR43-NLRP3-MAVS-type I IFN axis, while indole-3-propionic acid supplementation lowers viral load and systemic inflammation. Immune and barrier pathways coupled to these shifts include TLR3/4/7, NF-κB, cGAS-STING, JAK-STAT, PI3K-AKT-mTOR, NLRP3 inflammasome, chemokines (CCL5, CXCL10, MCP-1), and tight junction proteins (Occludin, Claudin-1, ZO-1), together shaping type I IFN signaling, inflammatory tone, and epithelial integrity in IAV models. Several mechanisms in this section are data-supported, for example, supplementation with indole-3-propionic acid reduces viral load and inflammation, microbiota-derived acetate enhances anti-IAV immunity via the GPR43-NLRP3-MAVS-type I IFN axis, and segmented filamentous bacteria restrain proinflammatory programs in alveolar macrophages. By contrast, many findings on compositional shifts and outcomes are correlational and require functional validation and causal confirmation; confounders such as antibiotics, diet, and concurrent medications may limit generalizability.

Across microbiome-IAV studies, several confounders merit explicit control. Antibiotic exposure can blunt microbiome-mediated immunity and vaccine responses in humans, potentially masking gut–lung axis effects; documenting timing and implementing washout or stratification are essential. Diet strongly shapes SCFAs and indoles; in mice, dietary modulation outperformed FMT in restoring the microbiome, underscoring the need for standardized chow or dietary logs and fiber content reporting. Concurrent medications and supportive care may shift taxa and metabolites; prior infection and vaccination history likewise alter immune tone. Comorbidities, age, sex, and host genetics contribute to baseline heterogeneity, warranting predefined subgroup analyses. Design and technical factors-sampling windows, diurnal variation, stool vs. mucosal sampling, mouse vendor/cage effects and co-housing, FMT donor variability, as well as 16S region selection, extraction kits, depth and batch-should be harmonized where possible. Mitigations include preregistered protocols, detailed metadata capture, absolute/quantitative profiling, co-primary functional readouts-, and sensitivity analyses to strengthen causal interpretation.

## 3 TCM diagnostic and therapeutic framework for IAV

In TCM, influenza A (21) falls under the categories of “epidemic qi,” “pestilential disease,” “seasonal epidemic cold,” and “seasonal epidemic toxin,” characterized by abrupt onset, fulminant course, rapid transmission and transformation, easy spread, and high contagion. Seasonal epidemic cold often results from the six exogenous pathogens compounded by seasonal evils. It involves both timely seasonal qi and untimely pathogens, with wind predominating, sometimes combined with cold, heat, dampness, or summer - heat, and prone to transformation into fire. Hence, onset is acute and transmission rapid: from the beginning the pathogen progresses from the defensive (wei) to the qi level, lodging between exterior and interior, with simultaneous involvement of wei and qi; exterior signs have not resolved while interior heat is already exuberant, transmitting to the nutritive (ying) level and entering the interior. Lei Zheng Zhi Cai: Shangfeng (*Classified Treatment of Syndromes: Wind - Induced Illness*) records that seasonal epidemic colds arise from external wind combined with cold, heat, summer - heat, and dampness, with a pathomechanism of external invasion leading to dysfunction of the lung and defensive systems; the main therapeutic principle is to dispel wind, disseminate the lung, and release the exterior. Bingyin Maizhi: Shanghan Zonglun (*Etiology and Pulse Diagnosis: General Treatise on Cold Damage*) emphasizes the invasion of untimely qi and pestilential qi; epidemic toxins enter through the mouth and nose, may transform according to the patient’s constitution, and transmit internally to the zang - fu. It advocates pattern - based treatment and the principle “when the pathogen is in the skin, induce sweating to release it.” Zhu Bing Yuan Hou Lun (*General Treatise on the Causes and Symptoms of Diseases*) first proposed methods for preventing pestilence: “Seasonal epidemic diseases arise from discordant seasons and loss of moderation in warmth and coolness; people contract aberrant qi and become ill, with high mutual contagion; therefore, take medicine in advance to prevent it.” Regarding population - level evidence on patterns and efficacy, a syndrome statistics study (*n* = 345) found wind - heat invading the defensive level to be most common, with damp - heat, qi deficiency, and mixed cold - damp also present (22). An analysis of 499 mild cases likewise indicated that most exhibited wind - heat invading the defensive level with dampness; those with shorter courses were dominated by wind - cold constraining the exterior, and among patients not receiving antiviral drugs, wind - heat with dampness still predominated (23). In children with springtime influenza A, wind - heat invading the defensive level and heat - toxin attacking the lung were the main patterns, with rapid evolution; dampness and stagnation readily complicated the course, transforming into interior heat (24). Over the centuries, physicians have employed major therapeutic methods such as inducing sweating, acrid - cool dispersal, harmonizing, attacking pathogens, and tonifying the spleen to prevent and treat influenza. Building on Zhang Zhongjing’s six - channel differentiation for exogenous disorders, Professor Zhou Zhongying (25) proposed concurrent resolution of exterior and interior with the combined use of four methods - sweating, harmonizing, clearing, and purging - for the pattern - based management of seasonal epidemic colds.

The exterior-interior relationship between the lung and large intestine is a fundamental theory within TCM’s zang - fu doctrine, originating in the Huangdi Neijing. The Ling Shu: Jingluo chapter describes the meridian pathways of the lung and intestines: the Hand Taiyin Lung channel and the Hand Yangming Large Intestine channel are internally - externally related via the meridians, coordinating physiologically and influencing each other pathologically. In terms of qi dynamics, the lung governs diffusion and downbearing, while the large intestine governs transmission of waste; failure of lung qi to descend can cause constipation, and heat accumulation in the large intestine can counterflow upward to the lung, inducing cough and dyspnea. As stated in Ling Shu: Four Seasons Qi, “When the abdomen frequently rumbles, qi surges to the chest, and dyspnea prevents prolonged sitting, the pathogen resides in the large intestine.” Guided by this theory, numerous formulas have been created to “treat the lung via the intestines” and to “treat the lung and intestine together.” Treating seasonal epidemic colds from the perspective of lung - intestine co-treatment is grounded in the exterior-interior relationship of the lung and large intestine and the warm - disease clinical rule that “epidemic toxin attacking the lung often implicates the intestine.” This lung - intestine co-treatment theory converges with modern research on the gut-lung axis, supported by generations of clinical practice and foundational studies, and continues to be applied and explored. Growing evidence indicates that dietary interventions, fecal microbiota transplantation (FMT), microbial therapeutics (probiotics and prebiotics), and Chinese medicines can effectively improve lung function and prevent and control influenza by modulating the gut microbiota and its metabolites (26–29). In interventional research, a randomized, grouped study (*n* = 186) comparing Jingyin Shuangjie San granules with oseltamivir phosphate capsules showed that after 3 days of treatment, the former achieved shorter times to complete defervescence and relief of fever, cough, and myalgia, greater reductions in TCM symptom scores, and a higher overall clinical efficacy rate (30). Among adults with wind - heat complicated by dampness, modified Yinqiao San and oseltamivir demonstrated broadly comparable efficacy; in the combined Chinese - Western medicine group, the median times to clinical cure and defervescence were 60 and 36 h, respectively, with a 97% cure rate by TCM syndrome criteria (31). Animal experiments also support the anti-inflammatory and antiviral potential of certain formulas: Haoqin Qingdan Decoction increased survival in IAV - infected mice, reduced lung index and viral titers, and attenuated lung tissue injury, primarily involving the JAK-STAT pathway (32); Maxing Shigan Decoction mitigated IAV - induced histopathological damage, reduced viral load and inflammatory cytokines, with mechanisms related to macrophage miR-1260/sema3a regulation of the PI3K/AKT/mTOR pathway (33).

The TCM exterior-interior relationship and “treating the lung via the intestine” constitute a conceptual framework with biological plausibility; modern support largely derives from animal models showing measurable improvements in gut barrier, chemokines, and inflammatory pathways, and still requires functional and causal validation in human populations. Overall, the traditional concept of treating the lung and intestine together is intrinsically congruent with the modern gut-lung axis at physiological and pathological levels, providing dual support in theory and mechanism for TCM pattern - based management of influenza A. It also suggests future refinement focusing on functional taxa and metabolites, optimization of intervention timing and dosing, and greater precision in formula composition.

## 4 Synergy of TCM and gut-lung axis regulation in IAV therapy

**FIGURE 1:**
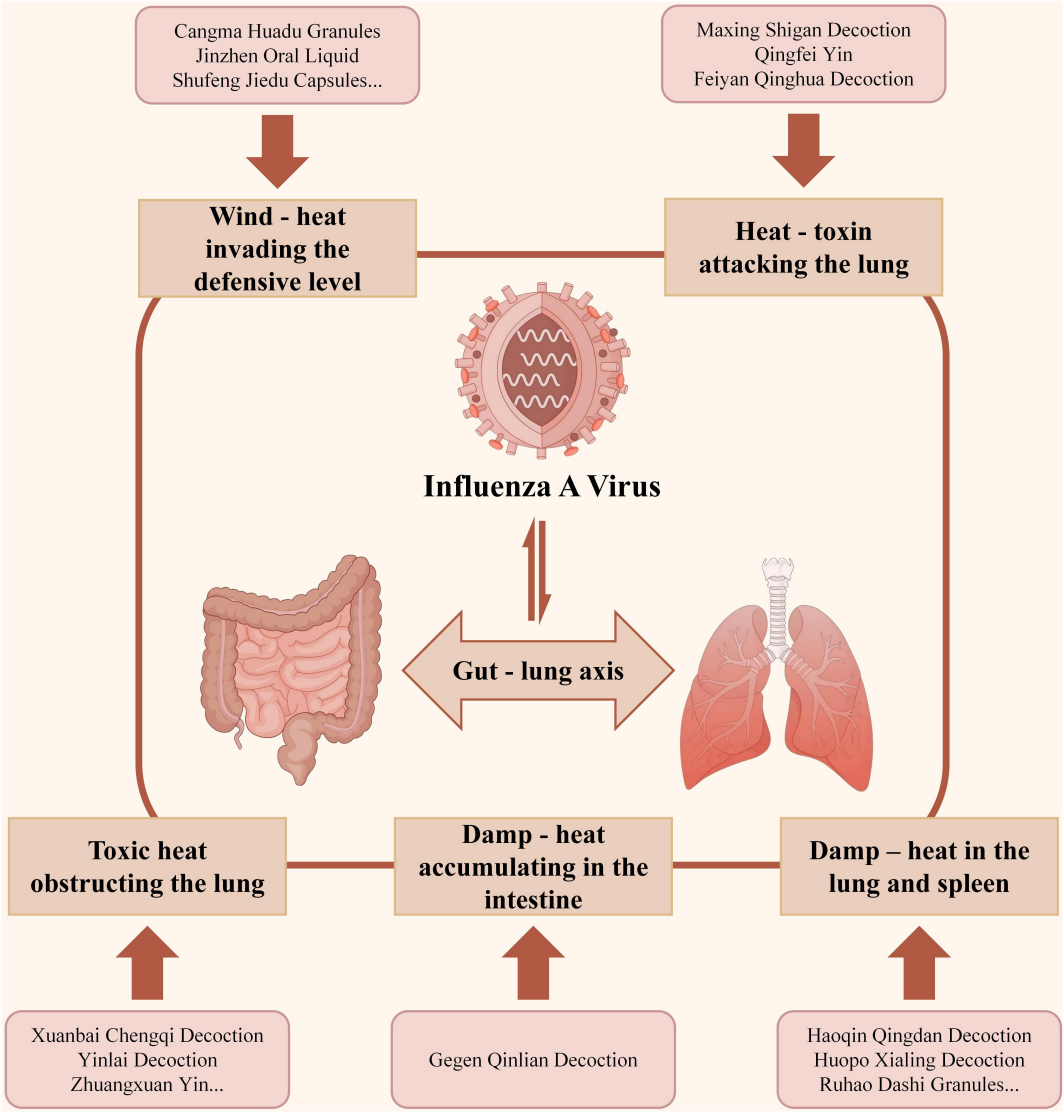
Traditional Chinese Medicine (TCM) syndrome patterns of Chinese herbal formulas for influenza A virus (IAV) treatment together with the gut-lung axis involved.

Chinese herbal formulas have a long history in treating exogenous disorders, with an emphasis on holistic pattern differentiation and treatment. Research on IAV indicates that Chinese formulas act through coordinated multi - component effects on multiple targets (including immune modulation, anti-inflammation, antiviral activity, and maintenance of microbiome homeostasis). Accordingly, therapeutic paradigms have shifted from the conventional “one drug - one target - one disease” to “multi - target - multi - component therapy” (Table 1) (34–37). Drawing on classical and contemporary clinical reasoning, modern gut-lung axis - based studies can be reviewed across patterns such as wind - heat invading the defensive level, heat - toxin attacking the lung, toxic heat obstructing the lung, damp - heat accumulating in the intestine, and damp - heat in the lung and spleen (Figure 1) (38, 39). This section presents mechanistic and preclinical evidence for TCM formulas, emphasizing integrated regulation of microbiota, metabolites, and immune pathways.

**TABLE 1:** Composition and mechanism of Chinese herbal formulas used to treat influenza A virus (IAV).

TCM syndrome pattern	Chinese herbal formulas	Composition	Main mechanism	References
Wind - heat invading the defensive level	Cangma Huadu Granules	*Atractylodes lancea* (Thunb.) DC., *Ephedra intermedia Schrenk et C. A. Mey.*, *Pogostemon cablin* (Blanco) Benth., *Belamcanda chinensis* (L.) DC., *Sophora tonkinensis* Gagnep., *Acacia catechu* (L. f.) Willd., *Physalis alkekengi* L. var. *Franchetii* (Mast.) Makino, *Glycyrrhiza uralensis* Fisch.	IL-1β↓, TNF-α↓, MDA↓, IL-10↑, SOD↑, GSH-PX↑, Bcl-2/Bax/caspase-3↓, *Bifidobacterium*↑, *Parasutterella*↑, *Bacteroides*↑, *Faecalibaculum*↑	(40)
	Jinzhen Oral Liquid	Bovis Calculus, *Fritillaria ussuriensis* Maxim., Goral Horn, *Rheum palmatum* L., *Scutellaria baicalensis* Georgi, *Glycyrrhiza uralensis* Fisch., Lapis Chloriti, Gypsum Fibrosum	TNF-α↓, IFN-γ↓, *Lactobacillus*↑, Clostridia_UCG-014↓, *Ruminococcus*↓	(41)
	Shufeng Jiedu Capsules	*Polygonum cuspidatum* Sieb. et Zucc., *Forsythia suspensa* (Thunb.) Vahl, *Glycyrrhiza uralensis* Fisch., *Verbena officinalis* L., *Isatis indigotica* Fort., Patrinia scabiosaefolia, *Bupleurum chinense* DC., *Phragmites communis* Trin.	TNF-α↓, IL-6↓, Firmicutes↑, Lachnospiraceae↑, Bacteroidetes↓, Bacteroidaceae↓, ZO-1↑, Occludin↑, LPS↓, cGAS↓, p-STING↓, p-NF-κB↓	(42)
	Qin - Qiao Xiaodu Formula	*Scutellaria baicalensis* Georgi, *Forsythia suspensa* (Thunb.) Vahl, *Mentha haplocalyx* Briq., *Platycodon grandiflorum* (Jacq.) A. DC., *Glycyrrhiza uralensis* Fisch.	IL-1α↓, IL-4↓, IL-12 (P70)↓, TNF-α↓, *Gemmiger*↑, *Anaerofustis*↑, *Adlercreutzia*↑, *Streptococcus*↑, *Dehalobacteriu*↓, *Burkholderia*↓, *Prevotella*↓, *Butyrimimonas*↓, *Delftia*↓, SCFAs metabolism↓, carbohydrate metabolism↑	(43)
	Tanreqing Capsules	*Scutellaria baicalensis* Georgi, Bear bile powder, Goral horn, *Lonicera japonica* Thunb., *Forsythia suspensa* (Thunb.) Vahl	Firmicutes↑, Actinobacteria↑, Bacteroidota↓, Proteobacteria↓, unclassified_f_Lachnospiraceae↑, Bacteroides↓, *Eubacterium*↓, *Phocaeicola*↓, Occludin↑, ZO-1↑, Muc2↑	(44)
	Jiajian Yinqiao San	*Lonicera japonica* Thunb., *Forsythia suspensa* (Thunb.) Vahl, *Chrysanthemum indicum* L., *Pogostemon cablin* (Blanco) Benth., *Atractylodes lancea* (Thunb.) DC.	TNF-α↓, IL-6↓, IL-17↓, TLR3↓, TLR4↓, TLR9↓, αvβ3↓, avα4 ↓, sIgA↑	(45)
	Rujin Jiedu Decoction	*Coptis chinensis* Franch., *Scutellaria baicalensis* Georgi, *Phellodendron chinense* Schneid., *Gardenia jasminoides* Ellis, *Platycodon grandiflorum* (Jacq.) A. DC., *Glycyrrhiza uralensis* Fisch.	IL-6↓, IL-1β↓, TNF-α↓, IFN-β↓, MCP-1↓, IP-10↓, IL-10↑, TGF-β↑, Bifidobacteriaceae↑, *Lachnoclostridium*↑, Lachnospiraceae_UCG-006↑	(46)
Heat - toxin attacking the lung	Maxing Shigan Decoction	*Ephedra intermedia Schrenk et C. A. Mey.*, *Prunus armeniaca* L. var. *ansu* Maxim., Gypsum Fibrosum, *Glycyrrhiza uralensis* Fisch.	CCL4↓, CCL2↓, CCL 5↓, CXCR2↓, CCR6↓, CXCR3↓, Actinobacteriotalevel↑, Desulfobacterota↑, Planctomycetota↓	(47)
	Qingfei Yin	*Ephedra intermedia Schrenk et C. A. Mey.*, *Prunus armeniaca* L. var. *ansu* Maxim., Gypsum Fibrosum, *Scutellaria baicalensis* Georgi, *Bombyx mori* Linnaeus, *Polygonum cuspidatum* Sieb. et Zucc., Oldenlandia diffusa (Willd) Roxb., *Houttuynia cordata* Thunb., *Trichosanthes kirilowii* Maxim., *Platycodon grandiflorum* (Jacq.) A. DC., *Forsythia suspensa* (Thunb.) Vahl, *Glycyrrhiza uralensis* Fisch.	MAPK↓, TNF-α↓, JAK-STAT↓, *Coprococcus*↑, *Ruminococcus*↑, *Lactobacillus*↑, *Prevotella*↑, *Escherichia*↓, *Parabacteroides*↓, *Butyricimonas*↓, *Anacrotruncus*↓	(52)
	Feiyan Qinghua Decoction	*Ephedra intermedia Schrenk et C. A. Mey.*, *Prunus armeniaca* L. var. *ansu* Maxim., Gypsum Fibrosum, *Glycyrrhiza uralensis* Fisch., *Bupleurum chinense* DC., *Scutellaria baicalensis* Georgi, *Rheum palmatum* L., *Fagopyrum esculentum* Moench, *Morus alba* L., *Pheretima aspergillum* (E. Perrier).	Lachnospiraceae_NK4A136↑, *Roseburia*↑, acetate↑, T-SOD↑, GSH-PX↑, MDA↓, Muc2↑, Occludin↑, Claudin-1↑, ZO-1↑	(53)
Toxic heat obstructing the lung	Xuanbai Chengqi Decoction	Gypsum Fibrosum, *Rheum palmatum* L., *Trichosanthes kirilowii* Maxim., *Prunus armeniaca* L. var. *ansu* Maxim.	IL1β↓, IL-6↓, TNF-α↓, TLR4↓, TLR7↓, MyD88↓, p65 NF-κB↓, Enterobacteriaceae↓, Proteus↓, Firmicutes↑, Lachnospiraceae↑	(54)
	Modified Xuanbai Chengqi Decoction	Gypsum Fibrosum, *Rheum palmatum* L., *Trichosanthes kirilowii* Maxim., *Prunus armeniaca* L. var. *ansu* Maxim., *Ephedra intermedia Schrenk et C. A. Mey.*, *Scutellaria baicalensis* Georgi, *Fritillaria thunbergii* Miq., *Morus alba* L., *Ilex rotunda* Thunb., *Glycyrrhiza uralensis* Fisch.	TNF-α↓, IL-1β↓, IL-6↓, Occludin↑, Claudin-1↑, ZO-1↑, LPS↓, Firmicutes/Bacteroidetes↓, *Akkermansia*↑, *Bifidobacterium*↑, *Bacteroides*↑	(56)
	Yinlai Decoction	*Lonicera japonica* Thunb., *Raphanus sativus* L., *Forsythia suspensa* (Thunb.) Vahl, *Scutellaria baicalensis* Georgi, *Peucedanum praeruptorum* Dunn, *Houttuynia cordata* Thunb., *Trichosanthes kirilowii* Maxim.	sIgA↑, IL-10↑, TNF-α↓, INF-γ↓	(57)
	Zhuangxuan Yin	*Sauropus spatulifolius* Beille, *Houttuynia cordata* Thunb., Lycopodiumclavatum, *Diospyros kaki* Thunb., *Spiranthes sinensis* (Pers.) Ames*[Neottia sinensis* Pers.*]*, *Glycyrrhiza uralensis* Fisch., *Citrus reticulata* Blanco, *Pinellia ternate* (Thunb.) Breit., *Ephedra intermedia Schrenk et C. A. Mey.*, *Schisandra chinensis* (Turcz.) Baill., *Atractylodes macrocephala* Koidz., *Hordeum vulgare* L.	TNF-α↓, IL-4↓, IL-6↓, IL-1β↓, p-p38 MAPK↓, p38 MAPK↓, Bacteroidales↑, Clostridia↓	(58)
Damp - heat accumulating in the intestine	Gegen Qinlian Decoction	*Scutellaria baicalensis* Georgi, *Coptis chinensis* Franch., *Pueraria lobata* (Willd.) Ohwi, *Glycyrrhiza uralensis* Fisch.	TNF-α↓, IL-1β↓, IL-6↓, INF-γ↓, *Akkermansia*↑, *Acetobacter*↑, C3↓, C3aR1↓, NLRP3↓, Caspase-1↓, ASC↓	(60)
Damp - heat in the lung and spleen	Haoqin Qingdan Decoction	*Artemisia annua* L., *Scutellaria baicalensis* Georgi, *Citrus reticulata* Blanco, *Pinellia ternate* (Thunb.) Breit., *Bambusa tuldoides* Munro, *Poria cocos* (Schw.) Wolf, *Citrus aurantium* L., *Isatis indigotica* Fort., *Isatis indigotica* Fort., *Glycyrrhiza uralensis* Fisch., Talc powder	IL-6↓, IL-1β↓, TNF-α↓, CCL2↓, CCL4↓, IP-10↓, IFN-β1↓, IRF3↓, HA↓, JAK1↓, JAK2↓, p-STAT1↓, p-STAT3↓, p-P65↓	(32)
	Qingfei Jiedu Formula	*Ephedra intermedia Schrenk et C. A. Mey.*, Gypsum Fibrosum, *Bupleurum chinense* DC., *Scutellaria baicalensis* Georgi, *Artemisia annua* L., *Atractylodes lancea* (Thunb.) DC., *Pogostemon cablin* (Blanco) Benth., *Verbena officinalis* L., *Glycyrrhiza uralensis* Fisch.	TNF-α↓, IL-1β↓, IL-6↓, CD4^+^↓, CD8^+^↑, CD45R↑, Verrucomicrobia↑	(62)
	Qianjin Sanhuang Decoction	*Ephedra intermedia Schrenk et C. A. Mey.*, *Scutellaria baicalensis* Georgi, *Astragalus membranaceus* (Fisch.) Bge. var. *Mongholicus* (Bge.) Hsiao, *Houttuynia cordata* Thunb.	TNF-α↓, IL-6↓, MAPK3↓, IL-1β↓, IL-10↑	(63)
	Huopo Xialing Decoction	*Pogostemon cablin* (Blanco) Benth., *Magnolia officinalis* Rehd. et Wils., *Pinellia ternate* (Thunb.) Breit., light red Indian Bread, *Prunus armeniaca* L. var. *ansu* Maxim., *Coix lacryma-jobi* L. var. *Mayuen* (Roman.) Stapf, *Amomum kravanh* Pierre ex Gagnep., *Polyporus umbellatus* (Pers.) Fries, *Glycine max* (L.) Merr., *Alisma orientale* (Sam.) Juzep., *Tetrapanax papyri fer* (Hook.) K. Koch	IL-1β↓, METTL3↓, NLRP3↓, ASC↓, pro-Caspase-1↓, cleaved Caspase-1↓, GSDMD-N↓	(64)
	Ruhao Dashi Granules	*Mosla chinensis* Maxim., *Amomum tsaoko* Crevost et Lemaire, *Magnolia officinalis* Rehd. et Wils., *Lonicera japonica* Thunb., *Artemisia annua* L., *Atractylodes lancea* (Thunb.) DC.	IL-6↓, TNF-α↓, sIgA↑, claudin-1↑, occludin↑, ZO-1↑, *Lactobacillus*↑, *Akkermansia*↑, *Faecalibaculum*↑, *Muribaculaceae*↓	(65)

### 4.1 Wind - heat invading the defensive level

The therapeutic principles are to dispel wind, release the exterior, clear heat, and resolve toxin, typically using Yinqiao San combined with Sangju Yin with modifications as the basic prescription (38). Cangma Huadu Granules regulate apoptosis - related proteins, reduce viral titers, inflammation, and oxidative stress, and improve gut microbiota composition and diversity (increasing Bifidobacterium, Bifidobacterium longum, and *Bacteroides*), thereby attenuating H1N1 - induced lung injury (40). Jinzhen Oral Liquid elevates Lactobacillus, reduces ClostridiaUCG-014 and Ruminococcus, and is accompanied by changes in small - molecule metabolites; it exerts anti-inflammatory and immunomodulatory effects to alleviate influenza - related lung injury (41). Shufeng Jiedu Capsules upregulate Firmicutes and Ruminococcaceae, downregulate *Bacteroidetes* and Bacteroidaceae, strengthen the intestinal barrier and reduce endotoxemia, and modulate the cGAS/STING/NF-κB pathway in lung tissue to mitigate inflammation, thereby alleviating H1N1 - induced lung damage (42). The Qin - Qiao Xiaodu Formula increases *Gemmiger*, *Anaerofustis*, *Adlercreutzia*, and *Streptococcus*, decreases *Dehalobacterium*, *Burkholderia*, and *Butyricimonas*, and promotes carbohydrate metabolism and the cyanoamino acid pathway; these changes correlate with improvements in mortality, weight loss, lung viral load, lung index, and tissue injury in IAV mice (43). Tanreqing Capsules increase microbial diversity, upregulate Firmicutes and Actinobacteria, downregulate *Bacteroidetes* and *Proteobacteria*, and at the genus level elevate unclassifiedf_Lachnospiraceae while decreasing *Bacteroides*, *Eubacterium*, and *Phocaeicola*; these shifts accompany improved body mass, reduced lung and intestinal injury, and higher survival in IAV - infected mice (44). Jiajian Yinqiao San suppresses IAV replication in the small and large intestines that leads to inflammatory lesions and enhances mucosal immunity in the lung and gut (45). Rujin Jiedu Decoction alleviates IAV - induced pneumonia by restoring short chain fatty acid producing bacteria (*Bifidobacteriaceae*, *Ruminococcaceae*, and *Ruminococcaceae* UCG-006) and downregulating inflammation - related signaling pathways (46).

### 4.2 Heat - toxin attacking the lung

The therapeutic principles are to clear heat and resolve toxin, diffuse the lung, and stop cough, with Maxing Shigan Decoction as the basic prescription. Multi-omics analyses show that Maxing Shigan Decoction reduces the abundance of *Flavisolibacter*, *Microvirga*, *Paenibacillus*, and *Solibacter* in the gut of IAV - infected mice, and decreases metabolites such as 2-nitrofuran, calcium oxalate, and xylazine; lung transcriptomics reveal diminished inflammatory cell infiltration, reflecting anti-inflammatory and immunomodulatory effects (47). Related studies observed downregulation of MCP-1 in lung tissue, correlated with MCP-1 expression in lung and colon, suggesting chemokine - mediated modulation (48); inhibition of CCL5 and CXCL10 with upregulation of Claudin-1 and Occludin in the intestine, improving lung - gut barrier function and inflammation (49); increased *Lactobacillus* with decreased Proteobacteria and Helicobacter, reduced lipopolysaccharide in serum and lung, and restoration of the intestinal mucosal barrier (50); increased relative abundance of *Firmicutes*, *Bacteroidetes*, and genera *Lactobacillus* and *Faecalibacterium*, decreased Verrucomicrobia, and modulation of CCL5 and CXCL10 production, together conferring protection (51). Qingfei Yin elevates *Faecalibacterium*, *Ruminococcus*, *Lactobacillus*, and *Prevotella*, prevents declines in *Escherichia*, *Parabacteroides*, *Butyricimonas*, and *Anaerotruncus*, downregulates inflammatory pathways including MAPK, TNFα, and JAK-STAT, reduces viral load and lung injury, and increases survival in H1N1 - infected mice (52). Feiyan Qinghua Decoction restores intestinal barrier integrity in IAV mice, increases LachnospiraceaeNK4A136group and Roseburia, elevates acetate and IFN-β, reduces viral load in lung tissue, and enhances antiviral responses, ameliorating lung and gut injury (53).

### 4.3 Toxic heat obstructing the lung pattern

The therapeutic principles are to resolve toxin and clear heat, drain the lung and activate the collaterals, with Xuanbai Chengqi Decoction as the basic prescription, modified as needed. Studies show that Xuanbai Chengqi Decoction modulates signaling pathways such as TNF, TCR, and NF-κB, reduces intestinal barrier leakage, and promotes pathogen clearance in IAV - infected mice, thereby protecting against lung and intestinal injury (54). In a co-infection model of IAV and *Streptococcus pneumoniae*, Xuanbai Chengqi Decoction increased the proportions of Firmicutes and Bacteroidetes, upregulated *Bacteroides*, Muribaculaceae, and *Ruminococcus*, corrected Foxp3/RORγt immune imbalance, and reduced lung - gut injury (55). Building on Xuanbai Chengqi Decoction, adding *Ephedra intermedia Schrenk et C. A. Mey.*, *Scutellaria baicalensis* Georgi, *Fritillaria thunbergii* Miq., *Morus alba* L., *Ilex rotunda* Thunb. and *Glycyrrhiza uralensis* Fisch., significantly reduced pulmonary bacterial load and inflammatory cytokines such as IL-6, TNF-α, and IL-1β in IAV - infected mice, increased tight junction protein expression, and improved survival, demonstrating efficacy and safety (56). In a food - stagnation model, Yinlai Decoction decreased sIgA and IFN-γ in lung tissue, upregulated IL-10, downregulated TNF-α and IFN-γ, improved mucosal immunity, and exerted anti-IAV effects (57). Zhuangxuan Yin ameliorated Bacteroidales abundance via inhibition of p38 MAPK signaling and reduced multiple inflammatory cytokines including TNF-α, IL-4, IL-6, and IL-1β, suggesting potential benefit for pediatric IAV pneumonia (58).

### 4.4 Damp - heat accumulating in the intestine pattern

The therapeutic principles are to clear heat, drain dampness, and harmonize the intestines and stomach, with Gegen Qinlian Decoction (GQD) as the basic prescription, modified as needed. Studies indicate that GQD alone or FMT - GQD increases *Akkermansia muciniphila*, *Desulfovibrio C21_c20*, and *Lactobacillus salivarius* while reducing *Escherichia coli*, inhibits the intestinal NOD/RIP2/NF-κB pathway, modulates inflammatory factor expression in mesenteric lymph nodes and serum, suppresses inflammatory differentiation of CD4+ T cells, lowers mortality, and alleviates lung inflammation in IAV - infected mice (59). Additional evidence suggests that GQD increases *Akkermansia* and *Acetobacter* in H1N1 - infected mice, inhibits the complement C3/NLRP3 inflammasome pathway, maintains metabolic and immune homeostasis, and reduces tissue injury (60). FMT - GQD illustrates the practical coupling among formula, microbiome, and host, although donor variability, long - term safety, and regulatory standards require further clarification.

### 4.5 Damp - heat in the lung and spleen pattern

The therapeutic principles are to clear heat and disseminate the lung, strengthen the spleen and resolve dampness, with Haoqin Qingdan Decoction as the basic prescription, modified as needed. Haoqin Qingdan Decoction downregulates Desulfovibrionaceae and Moraxellaceae, reduces intestinal barrier permeability and limits endotoxin translocation into the circulation, inhibits NP protein expression and the release of IL-6, IL-1β, and TNF-α, thereby markedly attenuating immune injury in the lungs and intestines of IAV - infected mice (61). The Qingfei Jiedu Formula increases the abundance of beneficial taxa such as Verrucomicrobia, regulates intestinal homeostasis, and improves survival in IAV - infected mice in a dose - dependent manner (62). Qianjin Sanhuang Decoction modulates signaling pathways including PI3K-Akt, TNF, and IL-17, alleviates colon shortening, and reduces lung index and immune injury in IAV - infected mice (63). Huopo Xialing Decoction increases *Lactobacillus* and decreases *Staphylococcus*, downregulates the pulmonary METTL3-NLRP3 (m6A) axis, reduces ASC, pro-caspase-1, cleaved caspase-1, and GSDMD-N, inhibits IL-1β expression, and improves lung injury in IAV - infected mice (64). Ruhao Dashi Granules elevate *Lactobacillus*, *Akkermansia*, and *Faecalibacterium* while reducing Muribaculaceae, suppress proinflammatory cytokines, and increase the expression of tight junction proteins Claudin-1, Occludin, and ZO-1, exerting therapeutic effects against IAV (65).

Research on Chinese herbal formulas for IAV has multidimensional limitations. Existing evidence largely derives from mouse IAV models coupled with multi-omics analyses (16S rRNA sequencing, metagenomics, metabolomics, and transcriptomics). Although these studies reveal multi-pathway synergy (microbiota modulation - barrier promotion - anti-inflammation - antiviral) and the intervention potential of the gut-lung axis, most are based on animal models and small - scale clinical studies, lacking multicenter randomized controlled trials with standardized preparations and quality control to validarte efficacy, safety, and translational applicability. Differences among formulas in herb sourcing, quality control, dosing, and timing affect reproducibility and comparability. The bidirectional regulatory mechanisms of gut barrier and chemokines observed for formulas such as Maxing Shigan Decoction vary across studies due to differences in microbiota resolution, time points, and sampling/sequencing standardization. While co-infection models offer a clinical - like scenario for “treating lung and intestine together,” modifications that increase formula complexity necessitate pharmacokinetic assessment and component standardization to avoid batch to batch fluctuations in efficacy. In addition to preparation and sampling heterogeneity, antibiotic/medication exposures, diet, comorbidities, age/sex, and mouse vendor/cage effects represent major confounders that should be prospectively recorded and controlled to improve reproducibility.

## 5 Synergistic mechanism of active ingredients of TCM in IAV therap

Active constituents of Chinese medicine in IAV - related studies exhibit multiple actions immunomodulatory, anti-inflammatory, antiviral, and remodeling of the intestinal micro ecosystem highlighting the gut-lung axis intervention potential of microbe - metabolite - immune synergy. This section focuses on mechanistic and preclinical evidence for active ingredients and polysaccharides and derives translational insights.

### 5.1 Monomeric constituents

Ginsenoside Rb1, primarily derived from *Panax quinquefolium* L., *Panax ginseng* C. A. Mey., and *Panax notoginseng* (Burk.) F. H. Chen, has immunomodulatory and anti-inflammatory effects. In one study (66), co-immunization of mice with an H1N1 influenza vaccine and Rb1 increased the abundance of *Akkermansiaceae* and *Muribaculaceae*, decreased IL-6 and TNF-α expression, elevated IgG and its subclasses, mitigated lung histopathology, and improved survival after viral challenge, suggesting dual roles as a vaccine adjuvant and a microecological modulator. Andrographolide, a diterpene lactone from *Andrographis paniculata*, possesses anti-inflammatory, anti-pathogenic, and immunomodulatory activities. In an IAV model, administration of an injectable preparation altered microbial diversity in the lung and gut, increasing putative beneficial taxa such as *Akkermansia*, *Parabacteroides goldsteinii*, *Defluviitaleaceae*, *Oscillospirales*, and the *Eubacterium coprostanol* genes group; binding of andrographolide to SERPINB2/PAI-2 was implicated as a potential anti-inflammatory and antiviral mechanism. Andrographolide significantly reduced mortality, weight loss, and lung viral titers, and improved pulmonary pathology (67). Berberine, an isoquinoline alkaloid extracted from *Coptis chinensis* Franch. and *Phellodendron chinense* Schneid., has antibacterial and anti-inflammatory effects. In IAV - infected mice, berberine increased the abundance of dominant taxa such as *Ruminococcaceae*, reduced the proportion of opportunistic pathogens, and suppressed excessive production of IFN-γ, IL-10, and CCL25 in serum and lung, thereby conferring protection (68).

### 5.2 Polysaccharides

#### 5.2.1 Houttuynia cordata polysaccharides

Houttuynia cordata polysaccharides enhance immunity and exert anti-inflammatory, antibacterial, antiviral, gut microbiota - modulating, and barrier - protective effects. They reduce the relative abundance of pathogenic genera such as *Vibrio* and *Bacillus*, suppress intestinal Toll-like receptors and IL-1β, elevate IL-10, improve intestinal homeostasis, and protect H1N1 - infected mice (69). They also lower concentrations of pulmonary proinflammatory cytokines/chemokines and reduce intestinal goblet cell numbers, increase intestinal sIgA and ZO-1, inhibit TLR4 and phosphorylated NF-κB p65 expression, decrease viral replication, mitigate lung - gut injury, and improve survival (70). Additionally, they decrease pulmonary CCL20 expression and modulate the CCR6+ Th17/CCR6+ Treg ratio, improving the Th17/Treg balance and lung injury (71). Further research shows that these polysaccharides inhibit excessive activation of intestinal complement C3a and C5a and the NLRP3 pathway, regulate the Th17/Treg balance, and thereby ameliorate acute pneumonia in co-infection with H1N1 and methicillin-resistant *Staphylococcus aureus* (72).

#### 5.2.2 *Ephedra sinica* stapf polysaccharides

*Ephedra sinica* stapf polysaccharides from the dried herbaceous stems of the *Ephedraceae* plant *Ephedra sinica* Stapf, possess immunosuppressive and antioxidant activities. They significantly increase the abundance of *Lactobacillales* and *Bifidobacteriaceae* while reducing *Enterococcaceae* in H1N1 - infected mice, suppress pulmonary TNF-α, IL-6, and IL-8 levels, lessen inflammatory cell infiltration, and improve virus - related immune responses, thereby exerting protective effects (73).

#### 5.2.3 Astragalus polysaccharides

Astragalus polysaccharides (APS) are natural immune enhancers that activate immune cells, promote antibody production, and modulate the gut microbiota. In a setting of two vaccine immunizations in mice (74), the dominant taxa after IAV infection were Colidextribacter, Erysipelotrichaceae, and a *Ruminococcaceae* outgroup. APS alleviated alveolar injury, upregulated Occludin and Claudin-1 in intestinal tissue, reduced serum TNF-α, increased survival, elevated IgG, IgG1, and IgG3 levels, and increased the frequency of CD8+ cells, thereby enhancing resistance to lethal infection.

### 5.3 Others

Baicalein tablets (a flavonoid from *Scutellaria baicalensis* Georgi) have anti-inflammatory and antiviral effects. One study (75) showed enrichment of intestinal Lactobacillus, significant downregulation of IL-12 p70, TNF-α, and IL-10 in lung tissue, and anti-IAV mechanisms involving TLRs and MAPK signaling. *Vanderbylia robiniophila*, first recorded in the Tang dynasty’s Newly Revised Materia Medica, has heat - clearing, detoxifying, anti-inflammatory, and antitumor actions. *Vanderbylia robiniophila* extract increased beneficial taxa such as *Alistipes* and *Alloprevotella*, influenced niacin/nicotinamide metabolism, reduced viral load and pathological damage in lung tissue, downregulated IL-6, TNF-α, and IFN-γ, and mitigated IAV - induced lung injury (76). A mixture of aromatic molecules derived from aromatic Chinese medicines (77) inhibited IAV replication and proinflammatory factor expression, improved colonic barrier injury, suppressed ROS and the NLRP3 inflammasome, corrected dysbiosis, and thereby increased survival.

Most findings here are preclinical (mouse) or vaccine-adjuvant contexts; claims such as andrographolide binding to SERPINB2/PAI-2 are inferred and require biophysical and intervention validation to confirm direct target engagement. Adjuvant-related increases in IgG subclasses and cellular immunity are reproducible, yet human translatability and dose-safety windows remain undefined. Microbiota shifts based on 16S relative abundance should be paired with metabolite and receptor-pathway functional tests to establish a causal “microbe-metabolite-immune” chain. Overall, active constituents predominantly demonstrate synergistic “microbiota modulation - barrier promotion - anti-inflammation - antiviral” effects in IAV mouse models and vaccine-adjuvant contexts, supporting “gut-lung axis” - based intervention strategies. As with Chinese herbal formulas, most studies on active constituents rely on 16S rRNA-level relative abundance analyses and short-term immune endpoints; there is an urgent need for absolute quantification, functional validation, metabolite profiling, and confirmation of causal chains to host targets. Attention should also be paid to component purity and batch consistency, pharmacokinetics and drug - drug interactions, and immunomodulatory risks across different ages, comorbidities, and vaccinated populations; establishing standardized quality and regulatory frameworks will improve reproducibility and clinical translation.

## 6 Conclusion

**FIGURE 2:**
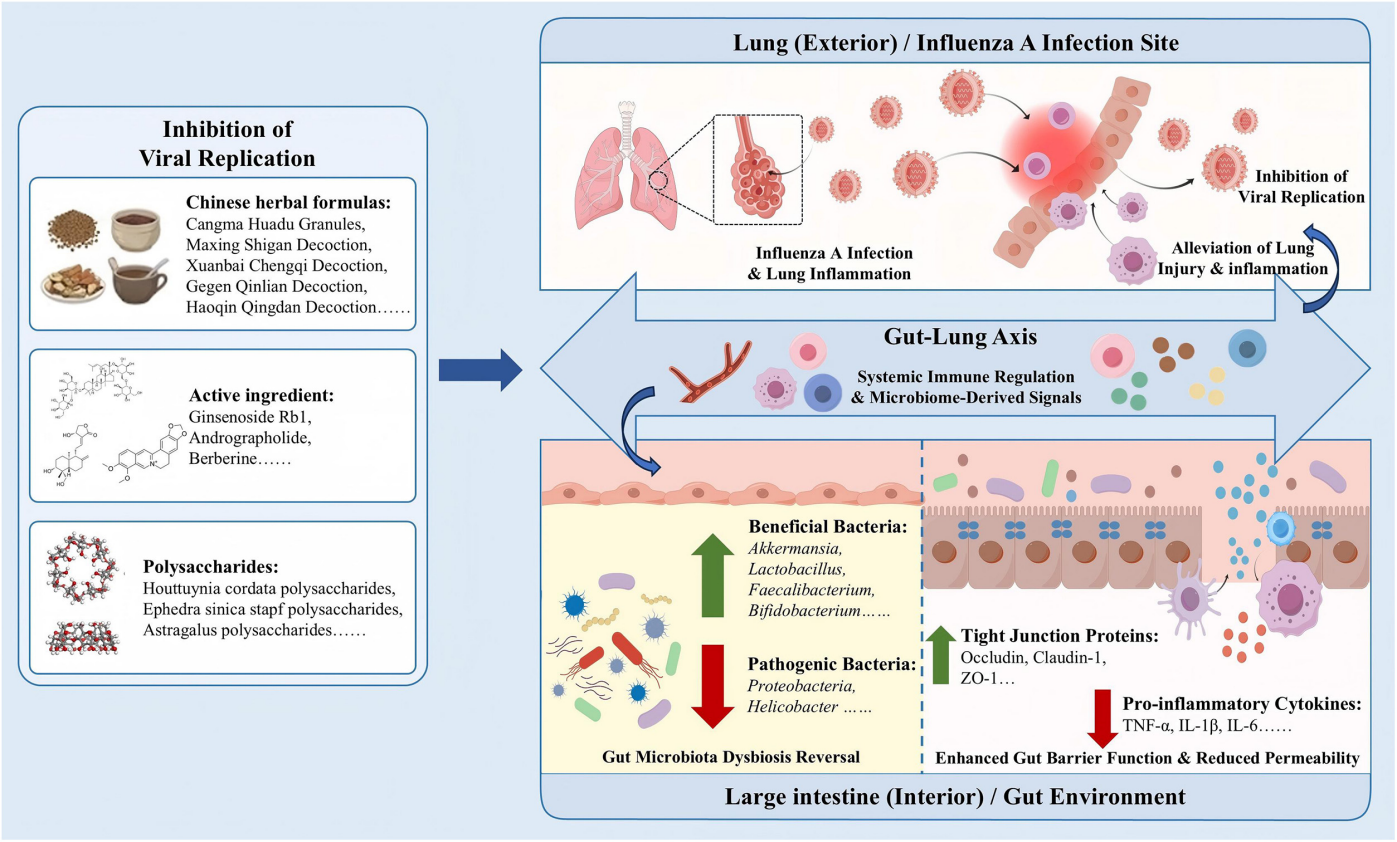
The “exterior-interior relationship of gut-lung” with modern immune-microbiome interaction.

Influenza A virus infection is among the most common respiratory diseases and, due to its high transmissibility and mortality, remains a major concern in public health. Starting from the TCM concept of the interior - exterior correlation between the lung and large intestine and the modern gut-lung axis, this article systematically reviews the TCM pattern - differentiation framework for IAV and its coupling with the microbe - metabolite - immune network. Chinese herbal formulas, single constituents, and polysaccharides generally exhibit multi - component, multi - target synergism in IAV - related studies, improving bidirectional injury of the lung and gut by reshaping gut microbiota structure and function, strengthening mucosal barriers, suppressing proinflammatory pathways, and promoting antiviral responses (Figure 2). These actions align with modern mechanistic evidence involving key signals such as TLR/NF-κB, cGAS-STING, JAK-STAT, PI3K-AKT-mTOR, the NLRP3 inflammasome, chemokine networks (CCL5, CXCL10, MCP-1), and type I interferons, alongside restoration of short chain fatty acid producing bacteria, indole metabolites, and tight junction proteins. Overall, formulas embody the comprehensive advantage of “treating the lung via the intestine” and “treating lung and intestine together,” while single constituents and polysaccharides provide clear molecular entry points for mechanistic dissection and formulation development, and show potential as vaccine adjuvants and in combination therapy with antivirals.

Compared with prior reviews centered on TCM antivirals or microbiota–immune interactions, our contribution lies in unifying the TCM exterior-interior doctrine with the gut-lung axis within a single framework, organizing evidence by TCM patterns, building explicit maps from formulas and active constituents to microbiota, metabolites, and immune nodes, and proposing mechanism-anchored translational pathways and trial designs. These elements provide actionable coordinates for precision application and standardized evaluation of TCM in IAV. It should be emphasized that current evidence is still dominated by IAV mouse models and small - sample clinical studies, with endpoints skewed toward short-term measures such as viral load, lung index, symptom scores, and inflammatory markers; translational applicability and long-term safety require higher - quality evidence. Many studies exhibit heterogeneity in sampling time points, analytical resolution, and preparation quality control, affecting reproducibility and comparability. Differences in efficacy across TCM patterns, ages, and comorbid populations, as well as optimal combination strategies with vaccines and antiviral drugs, also remain insufficiently defined.

Future work should focus on key strains and functional metabolites such as short-chain fatty acids, indoles, and bile acids, and their receptor pathways, to finely delineate the causal chain of “component - microbiota - metabolite - host targets,” and to define optimal intervention windows in terms of population, timing, dose, and formula. On the basis of standardized preparations and quality consistency, multicenter randomized controlled trials should be conducted using hard endpoints (hospitalization, complications, time to recovery, reinfection, and long-term outcomes), with stratified evaluation of efficacy and safety across different TCM patterns, ages, and comorbidities. Multi-omics and systems biology should be central, combined with humanized and germ-free models, absolute quantification, and high - throughput functional validation, to strengthen mechanistic interpretation and reproducibility. Mapping “pattern - microbiome - immune phenotype - metabolic markers” relationships will enable companion diagnostics and efficacy monitoring, advancing individualized formula composition and dose optimization. Synergy and interactions with vaccines and antivirals such as oseltamivir should be systematically assessed, with attention to pharmacokinetics, transporter/CYP effects, and risk management for excessive immunosuppression. Preclinical data suggest that selected TCM constituents may complement existing strategies through microbe-metabolite-immune modulation. For vaccination, ginsenoside Rb1 and APS have shown adjuvant-like effects in mice, enhancing IgG subclasses and cellular responses while reshaping beneficial taxa. For antivirals, gut-microbiota and network pharmacology analyses indicate potential synergy of Huangqin Su tablet with oseltamivir via attenuation of lung inflammation and stabilization of the gut barrier. Potential antagonisms should be considered: antibiotic co-use can blunt microbiome-mediated immune responses, over-suppression of inflammatory pathways may dampen vaccine immunogenicity if timing is suboptimal, and transporter/CYP interactions could alter antiviral exposure. Practically, any combination should prioritize timing, dose standardization, and monitoring of immune/barrier biomarkers to avoid excessive suppression or unintended interference. In parallel, raw material traceability, GMP-standardized manufacturing, quality fingerprints, and consistency evaluation should be advanced, and real-world studies conducted to verify effectiveness and safety in broad populations, thereby forming clinical intervention pathways that are regulatable, reproducible, and scalable to better support precise IAV prevention and control and public health needs.
